# Preparation and Parameter Optimization of Nanoscale Magnesium Hydroxide Colloids by the Electrical Spark Discharge Method under Ambient Conditions

**DOI:** 10.1021/acsomega.5c11909

**Published:** 2026-05-20

**Authors:** Meng-Yun Chung, Yi-An Lu, Yu-Ping Chou, Chaur-Yang Chang, Kuo-Hsiung Tseng

**Affiliations:** Department of Electrical Engineering, 34877 National Taipei University of Technology, Taipei 10608, TaiwanR.O.C.

## Abstract

In this study, an electrical spark discharge method was used under ambient temperature and pressure conditions to prepare magnesium hydroxide nanocolloids. The entire preparation procedure was conducted in DW to ensure that the manufacturing environment does not cause airborne nanoparticle dispersion. Two colloid preparation experiments were conducted by adjusting the parameters of peak current (IP) and pulse cycle time (*T*_P_). The results indicated that among all samples, the sample prepared with IP6 and *T*_P_ set to 70–70 μs exhibited the most favorable colloidal properties. Ultraviolet–visible spectrometry revealed a strong UV absorption peak at 192 nm, an absorbance value of 2.589, and zeta potential measurements showed a value of 35.2 mV. Transmission electron microscopy analysis indicated that the smallest particle size in the colloid was approximately 36.921 nm, with an almost spherical morphology, and that the lattice width of the particles was 0.237 nm.

## Graphical abstract

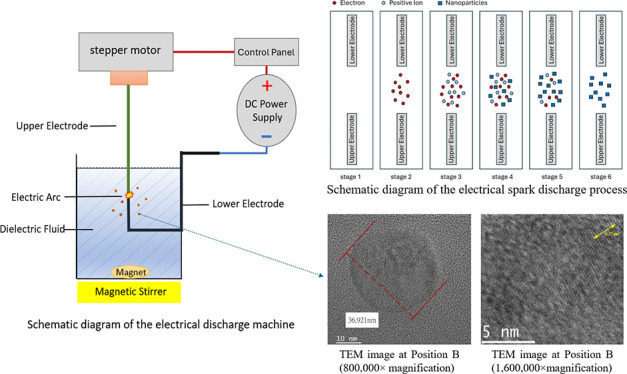

## 1 Introduction

Magnesium hydroxide nanoparticles are nontoxic, thermally stable, smoke-suppressive, antibacterial, antifungal nanoparticles that are widely applicable in various fields. In industrial settings, they are used as coating materials and flame retardants; in medical settings, they are used as antacids and laxatives;^1,2^ and in environmental settings, they are used in the treatment of acidic wastewater.^3–5^ From a thermal perspective, magnesium hydroxide exhibits good stability at low to moderate temperatures and undergoes dehydroxylation to form magnesium oxide (MgO) and water at elevated temperatures. Previous studies have reported that this transformation typically occurs at temperatures above approximately 350 °C, although the exact onset temperature depends on factors such as particle size, atmosphere, and heating conditions.^6–8^ This thermally induced transformation further underpins its widespread use in flame-retardant and fire-suppression systems.

Most of the commercially available magnesium hydroxide nanocolloids are synthesized using chemical methods.^9^ These methods often involve the addition of suspending agents to enhance material quality.^10,11^ Utilizing these agents in the food industry and in biomedical and environmental settings raises certain concerns regarding the potential adverse effects of their residues. Additionally, chemically synthesizing magnesium hydroxide nanocolloids may lead to nanoparticle dispersion in the manufacturing environment, which poses certain risks to human health and the environment. Evidence suggests that nanoparticles can cause pathological changes in lung tissues.^12^ Therefore, establishing an effective method for synthesizing magnesium hydroxide nanocolloids is a major concern in nanotechnology.

Applying the principle of electrical discharge machining to synthesize nanocolloids is known as the electrical spark-discharge method (ESDM). This technique utilizes the high temperature generated by spark discharge to vaporize the electrodes. The vaporized material then undergoes rapid quenching in a dielectric fluid (DF), forming nanoscale particles. Throughout this process, the colloid remains within the DF, ensuring that no nanoparticle dispersion occurs in the preparation environment. This ESDM-based technique is a physical method that has been successfully used for the preparation of silver, gold, and titanium nanocolloids as well as composite metal nanocolloids.^13–17^ These nanocolloids exhibit excellent suspension stability without requiring any suspending agents.^18^ Electrical discharge machining (EDM) is a nontraditional machining process that employs controlled electrical discharges between electrodes for material removal. When this discharge mechanism is utilized for nanomaterial synthesis instead of machining, the process is termed the electrical spark discharge method (ESDM). In this study, the term ESDM is adopted to emphasize its application in the nanocolloid preparation.

Magnesium is a highly reactive and flammable metal. When magnesium is placed in water, an exothermic reaction occurs, releasing hydrogen gas and forming magnesium hydroxide, as shown in eq 1. The smaller the particles of magnesium, the more intense the exothermic reaction, which may even trigger an explosion in certain cases. Therefore, to ensure safety, magnesium should be handled with caution. In the present ESDM process, however, the reaction is confined within deionized water, where the generated hydrogen remains primarily dissolved or as small bubbles in the liquid phase. Because of the absence of oxygen and the extremely low solubility of hydrogen in water, no explosion risk exists in the liquid phase.^19^

Potential safety concerns may arise primarily in large-scale or enclosed operations if hydrogen released from the liquid phase accumulates in the headspace and approaches flammable conditions. These risks can be mitigated using standard engineering controls such as adequate ventilation, vented reactor designs, hydrogen monitoring, and controlled off-gas handling. Furthermore, the controlled isolation of hydrogen using gas–liquid separation strategies enables its potential utilization as a clean energy carrier, thereby supporting a near zero-waste and environmentally benign synthesis concept.^20,21^

To the best of the authors’ knowledge, no studies have yet attempted to synthesize magnesium hydroxide nanocolloids by using the ESDM. More importantly, beyond its novelty, the proposed method directly addresses several inherent limitations of conventional chemical synthesis routes, including the reliance on chemical additives, the generation of secondary waste, and the risk of nanoparticle exposure. By employing a single-step, additive-free, and water-based physical process under ambient conditions, the ESDM provides a safer and more sustainable alternative for producing magnesium hydroxide nanocolloids with reduced environmental and health impacts. This study explored the optimal process parameters for preparing magnesium hydroxide nanocolloids through the ESDM. The nanocolloid samples produced in this study were analyzed using precision instruments to evaluate their performance. The results highlighted the technical contributions of the ESDM to the synthesis of magnesium hydroxide nanocolloids:

$$\mathrm{Mg}+2H_{2}O\to \mathrm{Mg}{\left(\mathrm{OH}\right)}_{2}+H_{2}↑$$ 1

## 2 Materials and Methods

### 2.1 ESDM-Based Nanocolloid Preparation

To prepare nanocolloids by using the ESDM, the high temperature generated by the spark discharge reaction is used to vaporize the surface material of the electrodes.^22–24^ The vaporized electrode material then undergoes rapid condensation in a DF, forming nanoparticles. This preparation procedure involves an electrical discharge machine, a DF, and two electrically conductive wires. These two wires are immersed in the DF and serve as the upper and lower electrodes, with the space between them termed the interelectrode gap (IEG). When the pulsed direct current power supply of the electrical discharge machine is connected to the upper and lower electrodes, maintaining the IEG within the microscale range enables a periodic spark discharge within the IEG. The efficiency of nanomaterial synthesis with the ESDM is closely related to the conditions of discharge within the IEG, which are influenced by factors such as the IEG size, residual molten slag within the IEG, insulation properties of the DF, open-circuit voltage, peak current (IP), pulse-on time (*T*_on_), and pulse-off time (*T*_off_).

During ESDM-based colloid preparation, the pulse cycle time (*T*_P_) is divided into *T*_on_ and *T*_off_, representing two discharge phases (Figure 1). Figure 2 depicts the voltage (*V*_IEG_) and current (*I*_IEG_) in the IEG. The ESDM discharge process is described as follows:

**Figure 1:**
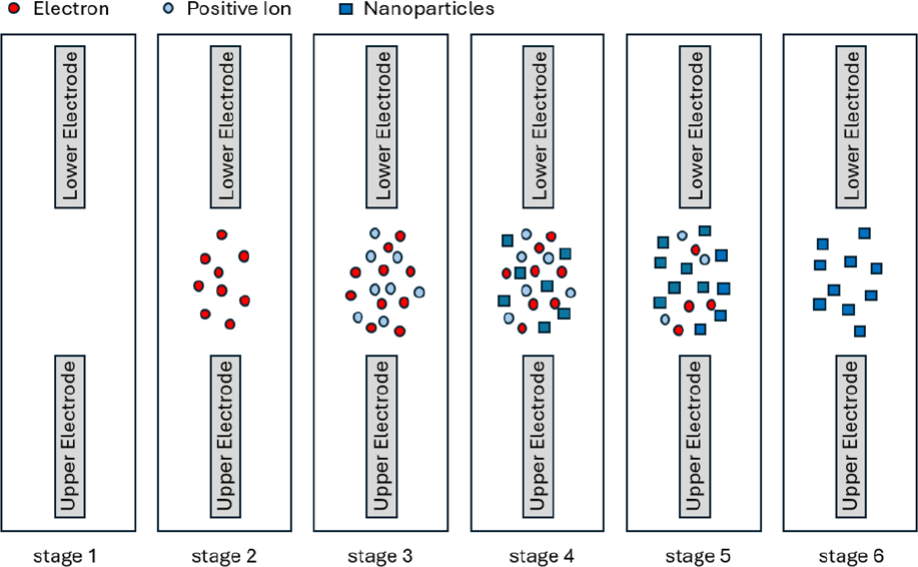
Electrode discharge conditions during ESDM-based nanocolloid preparation.

**Figure 2:**
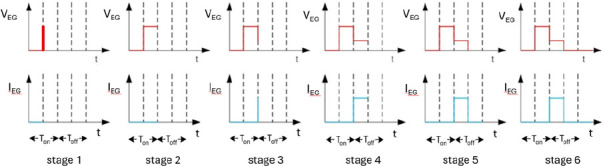
*V*_IEG_ and *I*_IEG_ during ESDM-based nanocolloid preparation.

In stage 1 (discharge preparation), the IEG remains in an insulated state, with *I*_IEG_ = 0.

In stage 2 (discharge initiation), the upper and lower electrodes gradually approach each other, and the electric field intensity in the IEG increases. As the electric field intensity increases, the insulating capability of DF within the gap decreases. Consequently, a small number of electrons begin to travel from the lower electrode to the upper electrode,^25,26^ gradually forming a discharge channel within the IEG.

In stage 3 (ionization), the electric field causes the electrons to collide at a high velocity with DF molecules within the IEG, which in turn causes a large number of DF molecules to dissociate into positive ions and free electrons. This process results in the formation of a narrow plasma column within the IEG. Breaking down the insulation of the DF leads to a sharp decrease in *V*_IEG_ and a rapid increase in *I*_IEG_, until it reaches its maximum.

In stage 4 (electrical spark effect), free electrons and positive ions accelerate under the influence of the electric field and collide with the upper and lower electrodes at a high velocity. These collisions generate electric sparks and a high temperature, which is sufficient to vaporize the surface material of the electrodes.^22–24^ The vaporized electrode material then undergoes rapid quenching in the DF, forming nanoparticles. During this stage, the DF remains in a low-resistance state, *I*_IEG_ remains at its maximum, and *V*_IEG_ remains constant.

In stage 5 (discharge termination), the electric field diminishes, and the resistance of the DF gradually increases, causing the current to decrease. The nanoparticles generated during the *T*_on_ period remained suspended in the DF.

In stage 6 (insulation recovery), the IEG returns to its insulated state, with both *V*_IEG_ and *I*_IEG_ dropping to zero. This insulation recovery phase was followed by another discharge cycle. This entire periodic discharge process yields the required nanocolloids.

Under the high-energy and strong electric-field conditions of the ESDM process, magnesium atoms are ionized into Mg^2+^ species, while water molecules dissociate into H^+^ and OH^–^ ions. The Mg^2+^ ions subsequently react with OH^–^ to form Mg­(OH)_2_ nanoparticles, and the generated H^+^ ions are reduced to H_2_ gas and released from the system.^27,28^ The proposed formation mechanism is schematically illustrated in Figure 3.

**Figure 3:**
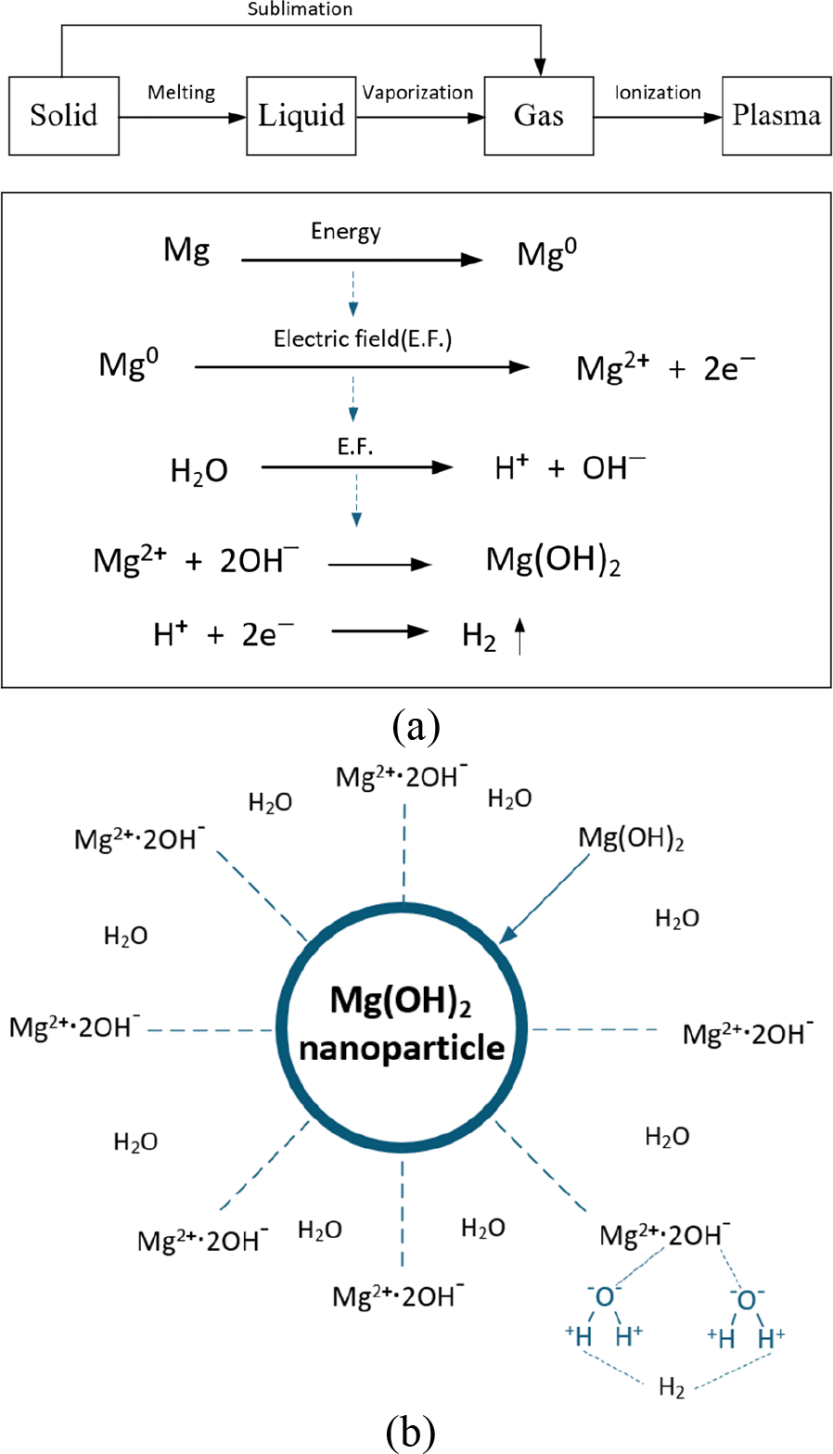
Proposed formation mechanism: (a) disassociation of Mg+; (b) suspension of nano-Mg­(OH)_2_.

### 2.2 Magnesium Hydroxide Nanocolloid Preparation System

Figure 4 depicts the setup used to prepare the magnesium hydroxide nanocolloids. This setup consists of a beaker containing deionized water (DW) and a magnetic stir bar placed on a magnetic stirrer. Both the upper and lower electrodes are magnesium wires, with the upper electrode secured by an electrical discharge machine clamp and the lower electrode secured by a 3D-printed fixture inside the beaker. During the colloid preparation process, the electrodes are immersed in the DW and connected to the positive and negative terminals of a pulsed power supply. A servo control system is used to regulate a servo motor by relying on current signals from the IEG to ensure that the electrode gap remains within an optimal range for spark discharge.^29^ The magnetic stirrer rotates the magnetic stir bar inside the beaker to promote uniform dispersion of nanoparticles within the DW.

**Figure 4:**
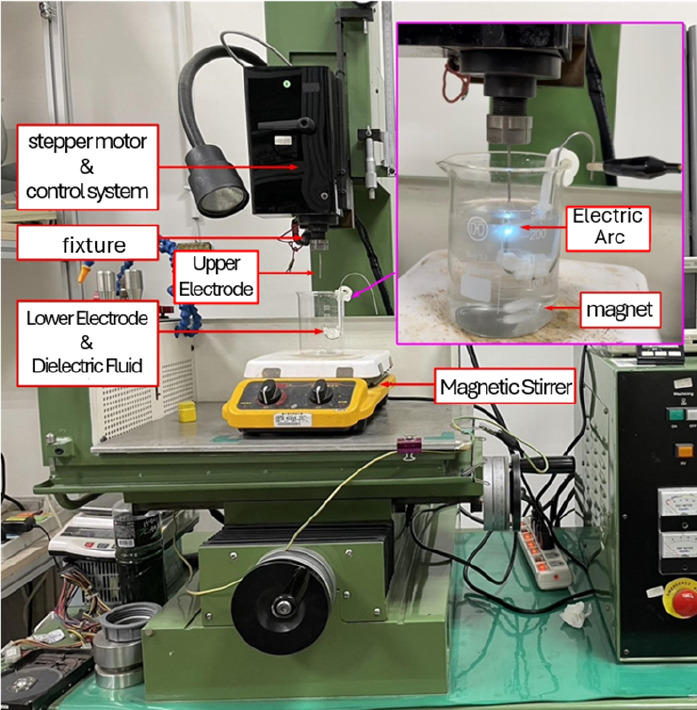
Setup used for the ESDM-based preparation of magnesium hydroxide nanocolloids.

### 2.3 Parameter Settings for Magnesium Hydroxide Nanocolloid Preparation Experiments

In terms of ESDM discharge characteristics, before spark discharge occurs during each *T*_on_, a short time delay is required for the electric field to break down the DF within the IEG. This short delay is referred to as the ignition delay time (*t*_D_). As *t*_D_ increases, the effective spark discharge time decreases. At a duty cycle (D) of 50%, a shorter *T*_on_ results in a higher switching frequency between *T*_on_ and *T*_off_, leading to a longer *t*_D_.^30^ Consequently, a longer *T*_on_ increases the cumulative spark discharge time. At the end of *T*_on_, the parasitic capacitance between the upper and lower electrodes stores residual energy in the IEG, which is proportional to *T*_on_. During *T*_off_, this parasitic capacitance releases this residual energy, which in turn enhances the insulation properties of the IEG. Increasing *T*_off_ enables the IEG to achieve greater insulation before the next discharge cycle, which in turn enhances the characteristics of spark discharge in the next cycle. In this study, magnesium hydroxide nanocolloid preparation experiments were conducted by varying IP and *T*_on_–*T*_off_ settings to determine the optimal preparation conditions for colloids.

The primary goal of the IP variation experiment was to ensure a long cumulative spark discharge time and maintain strong insulation at the end of the discharge cycle. The *T*_on_–*T*_off_ setting was fixed at 70–70 μs, and the IP setting was varied from IP1 to IP7 (2.4A, 5.6A, 8.4A, 11.8A, 17.2A, 25.0A, and 27.4A), with each condition used to prepare a unique colloid sample. The IP setting in an electrical discharge machining system represents a discrete control level rather than a linearly scaled current value, and the actual discharge current is governed by transient plasma conditions, dielectric properties, and internal power control mechanisms. The optimal IP setting for magnesium hydroxide nanocolloid preparation was determined by analyzing these colloid samples.

During the *T*_on_–*T*_off_ variation experiment, the optimal IP setting determined from the aforementioned experiment was used, along with 10 distinct *T*_on_–*T*_off_ settings, to prepare colloid samples. The characteristics of these colloid samples were examined to identify the optimal *T*_on_–*T*_off_ setting for magnesium hydroxide nanocolloid preparation. Table 1 presents the parameter settings, materials, and environmental conditions used in the colloid preparation process.

**Table 1:** Preparation Parameters for Magnesium Hydroxide Nanocolloids

experimental parameter	values
volume of the dielectric fluid	DI water: 200 mL
electrode diameter	pure magnesium wire (99.9%): 1.6 mm
T_on_–T_off_	10–10, 20–20, 30–30, 40–40, 50–50, 60–60, 70–70, 80–80, 90–90, 100–100 (μs)
peak current setting (IP)	IP1(2.4 A), IP2(5.6 A), IP3(8.4 A), IP4(11.8 A), IP5(17.2 A), IP6(25.0 A), IP7(27.4 A)
open voltage	140 V
preparation time	2 min
temperature	25 °C (room temperature)
atmospheric pressure	1 atm

### 2.4 Colloid Performance Analysis

In nanocolloid applications, factors such as particle concentration, size, suspension stability, morphology, and composition affect performance. In this study, the following instruments were used to examine the characteristics of the magnesium hydroxide nanocolloids prepared: an ultraviolet–visible (UV–vis) spectrometer (Thermo Helios Omega; Thermo Fisher Scientific, Waltham, MA, USA), a Zetasizer nanosystem (Zetasizer Nano ZS90; Malvern Instruments, Worcestershire, UK), a transmission electron microscope (JEM-2100F; JEOL, Tokyo, Japan), and an X-ray diffractometer (Panalytical Empyrean; Malvern Panalytical, UK). In the following, the measurement principles of these instruments are described.

Each substance exhibits a unique absorption spectrum because of its chemical structure. UV–vis spectrometry utilizes UV and visible light to illuminate the surface of the sample and obtain an absorption spectrum graph. In this graph, the horizontal axis represents the UV–vis absorption wavelength, and the vertical axis represents absorbance. The UV–vis absorption wavelength serves as a preliminary indicator of sample composition. According to the Beer–Lambert law, absorbance is directly proportional to concentration. Therefore, UV–vis absorbance can be used as an indicator of colloid concentration.

In colloidal solution systems, the repulsive force between particles is closely related to their surface potential. A greater repulsive force results in higher suspension stability. Zeta potential (*V*_ζ_) is an indicator of the charge state of particle surfaces and is widely used to evaluate particle suspension stability. In colloids, a larger absolute zeta potential (|V_ζ_|) indicates a stronger repulsive force between particles, which enhances colloidal suspension stability. A colloid is considered to have high suspension stability when its |*V*_ζ_| exceeds 30 mV. A Zetasizer is an instrument used to measure both zeta potential and hydrodynamic particle size.^31^ Zeta potential measurements are conducted using electrophoresis, in which an electric field drives the movement of particles with surface potential in the sample. In these particles, *V*_ζ_ can be calculated depending on their electrophoretic mobility and Henry’s equation. A Zetasizer utilizes dynamic light scattering technology to measure particle size. However, because this technology focuses on particles dispersed in liquid, Zetasizer measurements reflect the hydrodynamic size of particles. Particle size distribution can influence the measurement results.

Transmission electron microscopy (TEM) is commonly used to observe the particle size, morphology, and crystalline structure of nanomaterials.^32,33^ When combined with energy-dispersive X-ray spectroscopy (EDS), TEM can also analyze the elemental composition of the sample. TEM involves the use of a series of electromagnetic lenses to focus a high-energy electron beam onto a sample. As the electron beam penetrates the sample, it forms an image with a varying contrast.

X-ray diffraction (XRD) examines crystalline structure on the basis of Bragg’s law. XRD spectra are obtained from the diffraction phenomenon that occurs when X-rays interact with crystal atoms. In these spectra, information regarding a material’s crystalline structure is obtained from the positions, intensities, and widths of diffraction peaks. This crystalline structure is identified by comparing these XRD spectra against the International Centre for Diffraction Data database.

## 3 Results and Discussion

In this study, the ESDM was used to prepare magnesium hydroxide nanocolloids under ambient temperature and pressure conditions. Table 1 presents the parameter settings used in the colloid preparation process, with equal *T*_on_ and *T*_off_. Each colloid sample prepared was designated a name in the format of C-X-Y-Z, consisting of four components. Component C represents a colloid; component X indicates the duration of *T*_on_, measured in microseconds; component Y represents the preparation time, measured in minutes; and component Z denotes the IP setting. For example, in sample C-70–2m-2, the process parameters are *T*_on_ and *T*_off_ of 70 μs, a preparation time of 2 min, and an IP setting of IP2. Various magnesium hydroxide nanocolloid samples were prepared by varying the IP and *T*_on_–*T*_off_ settings. All samples were prepared over a fixed preparation time of 2 min. Their characteristics were examined using UV–vis spectrometry and Zetasizer measurements to determine their absorption spectra and particle size–number distribution and to calculate their *V*_ζ_ values. Finally, a colloid property analysis was conducted to identify the optimal parameters for preparing high-quality colloids.

### 3.1 Colloid Preparation Experiment with IP as a Variable

In this experiment, the *T*_on_–*T*_off_ setting was fixed at 70–70 μs and the IP setting was varied from IP1 to IP7 to prepare different colloidal samples. Figure 5 illustrates the UV–vis absorption spectra of these colloidal samples. The results indicated that the UV–vis absorption wavelengths of these samples ranged between 190 and 193 nm, with absorbance values ranging between 1.018 and 2.589.^34^ Table 2 presents a summary of the *V*_ζ_ values, particle size–number distribution, and absorbance analysis results of these colloidal samples. The data indicated that six colloidal samples had |*V*_ζ_| values greater than 30 mV, indicating their high suspension stability. At the lowest current setting (IP1), the measured zeta potential (−3.22 mV) was close to zero, suggesting that the discharge energy was insufficient to generate a measurable concentration of Mg­(OH)_2_ nanoparticles. Under this condition, the zeta potential may be mainly influenced by background interfacial signals in deionized water (e.g., trace nano/microbubbles or dissolved gases), which can result in apparent negative zeta potentials on the order of a few millivolts.^35,36^ Among these samples (Table 2), C-70–2m-2, C-70–2m-4, and C-70–2m-6 were identified as those with the smallest particle sizes. Figures 6, 7, and 8 depict the *V*_ζ_ values and particle size–number distribution analysis results of these samples. Among all seven colloidal samples outlined in Table 2, C-70–2m-6 exhibited the largest |V_ζ_| value, the highest absorbance, and the second-smallest particle size. Because of the colloidal properties of C-70–2m-6, this sample was identified as the optimal colloidal sample. Consequently, IP6 was identified as the optimal IP setting for the preparation of magnesium hydroxide nanocolloids in experiments involving IP as a variable factor.

**Figure 5:**
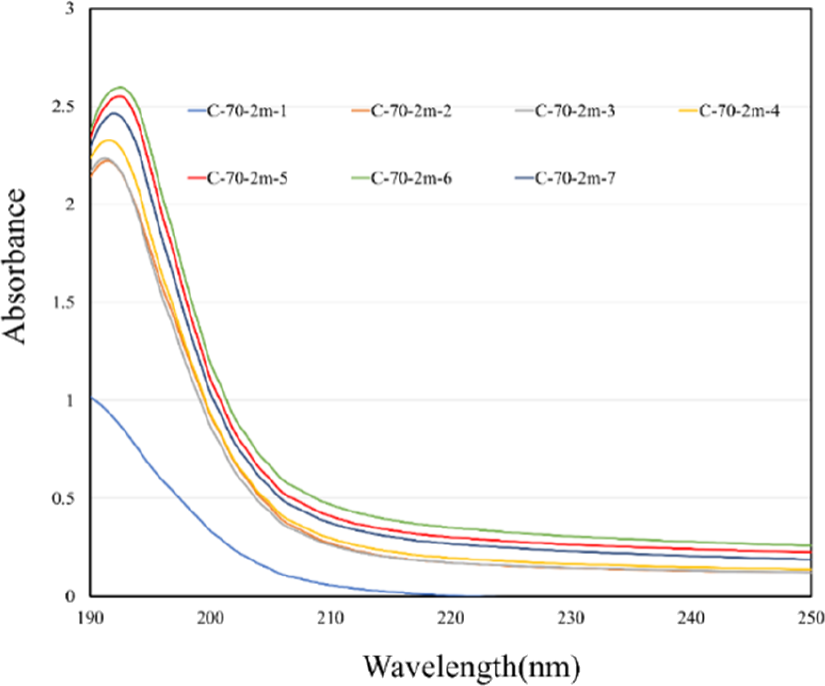
Absorption spectra of colloids prepared with seven IP settings at *T*_on_–*T*_off_ = 70–70 μs.

**Table 2:** Characteristics of Colloids Prepared with Seven IP Settings at *T*_on_–*T*_off_ = 70–70 μs

		particle size–number distribution		
colloid name	**V**_**ζ**_ (mV)	>100 nm (%)	particle size (d nm)	absorbance
C-70–2m-1	–3.22	100	87.03	1.018
C-70–2m-2	32.4	100	50.37	2.213
C-70–2m-3	34.2	100	70.59	2.232
C-70–2m-4	33.9	98.9	44.92	2.320
C-70–2m-5	34.3	99.9	51.76	2.544
C-70–2m-6	35.2	99.9	49.51	2.589
C-70–2m-7	30.8	100	52.66	2.463

**Figure 6:**
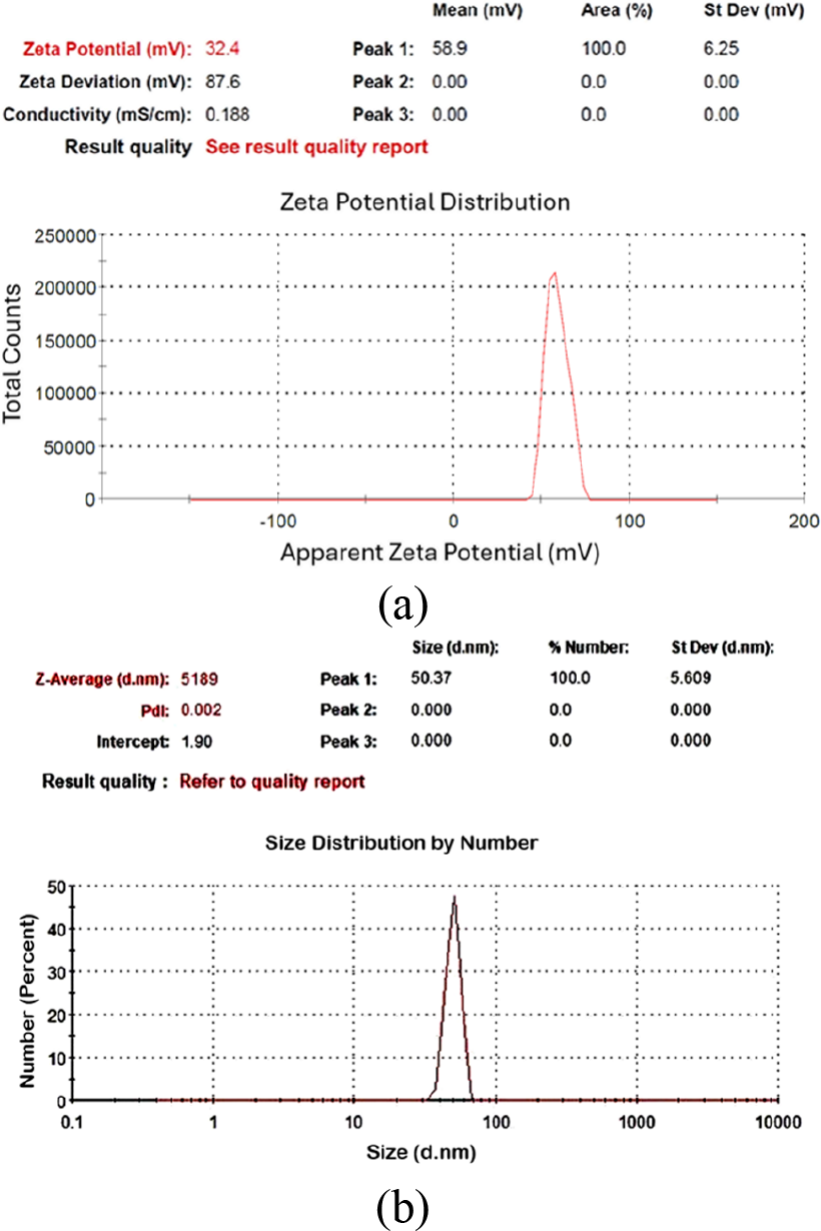
Analysis results of C-70–2m-2: (a) zeta potential, (b) particle size–number distribution.

**Figure 7:**
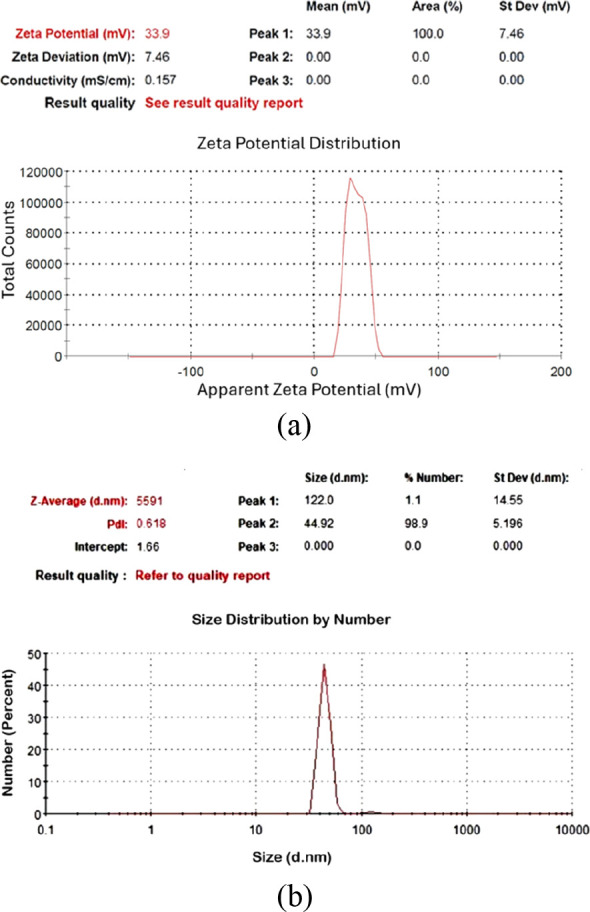
Analysis results of C-70–2m-4: (a) zeta potential, (b) particle size–number distribution.

**Figure 8:**
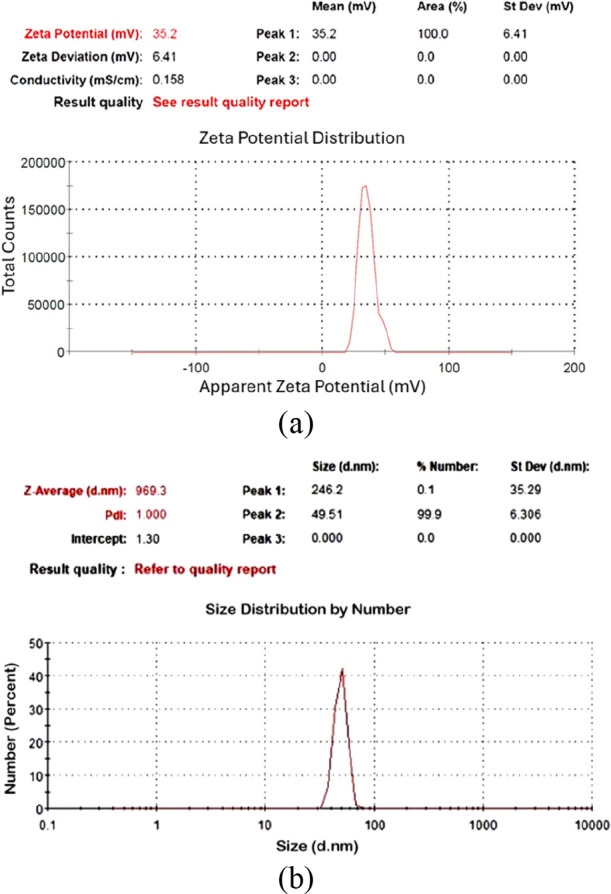
Analysis results of C-70–2m-6: (a) zeta potential, (b) particle size–number distribution.

### 3.2 Colloid Preparation Experiment with *T*_on_–*T*_off_ as a Variable

In this experiment, the IP setting was fixed at IP6 and the pulse cycle time parameter was varied to prepare different colloidal samples. A total of 10 pulse cycle time settings were used: 10–10, 20–20, 30–30, 40–40, 50–50, 60–60, 70–70, 80–80, 90–90, and 100–100 μs. Figure 9 illustrates the UV–vis absorption spectra of these colloidal samples. The results indicated that the UV–vis absorption wavelengths of these samples ranged between 193 and 197 nm, with absorbance values ranging between 2.258 and 2.742. Table 3 presents a summary of the *V*_ζ_ values, particle size–number distribution, and absorbance analysis results of these colloidal samples. The data revealed that only C-10–2m-6, C-70–2m-6, and C-90–2m-6 had |*V*_ζ_| values greater than 30 mV, indicating that only these three samples exhibited high suspension stability. Figures 10 and 11 depict the *V*_ζ_ values and particle size–number distribution analysis results of C-10–2m-6 and C-90–2m-6. Among the three colloidal samples with high suspension stability, C-70–2m-6 exhibited the highest |*V*_ζ_| value and the second-highest absorbance, with a particle size only slightly larger than those of the other two samples. Because of the colloidal properties of C-70–2m-6, this sample was identified as the optimal colloidal sample. Consequently, 70–70 μs was identified as the optimal *T*_on_–*T*_off_ setting for the preparation of magnesium hydroxide nanocolloids in experiments involving *T*_on_–*T*_off_ as a variable factor.

**Figure 9:**
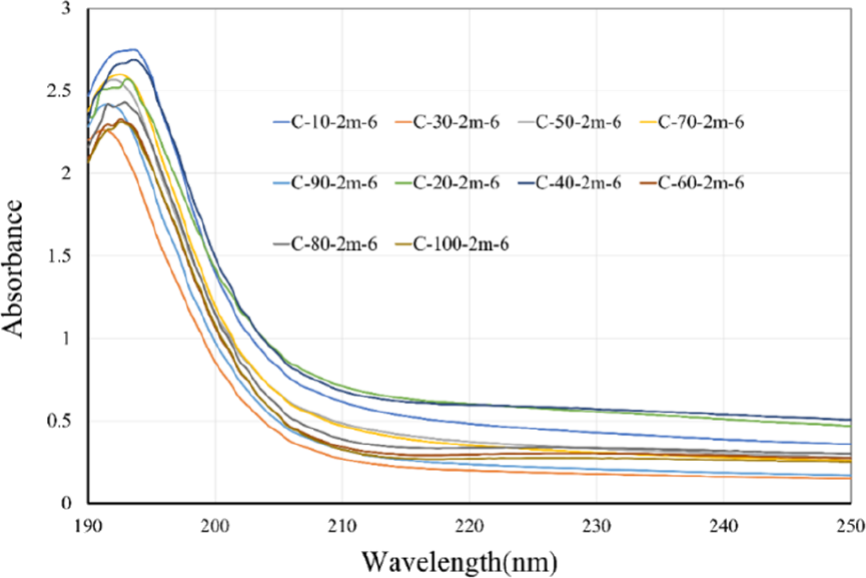
Absorption spectra of colloids prepared with different pulse cycle times with IP6.

**Figure 10:**
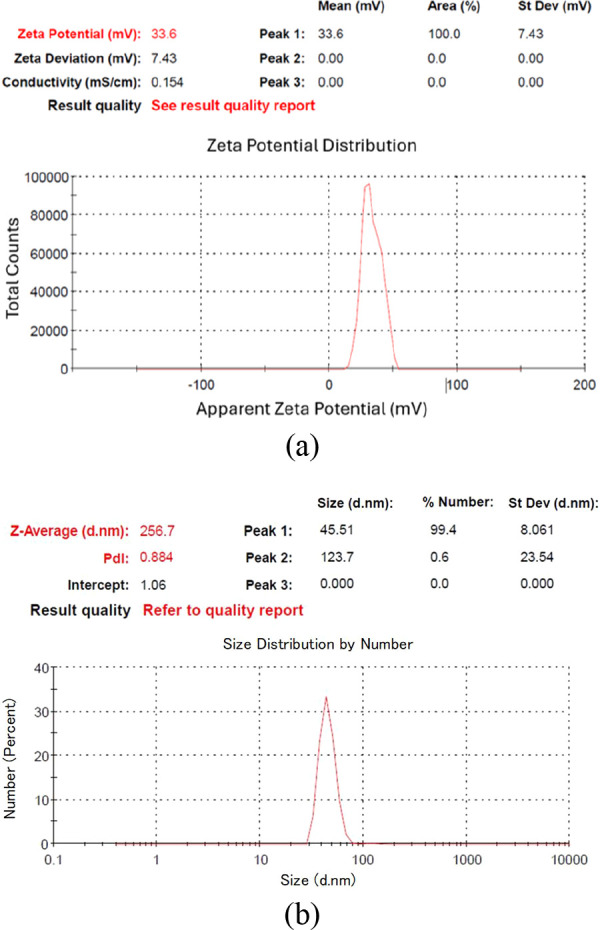
Analysis results of C-10–2m-6: (a) zeta potential, (b) particle size–number distribution.

**Figure 11:**
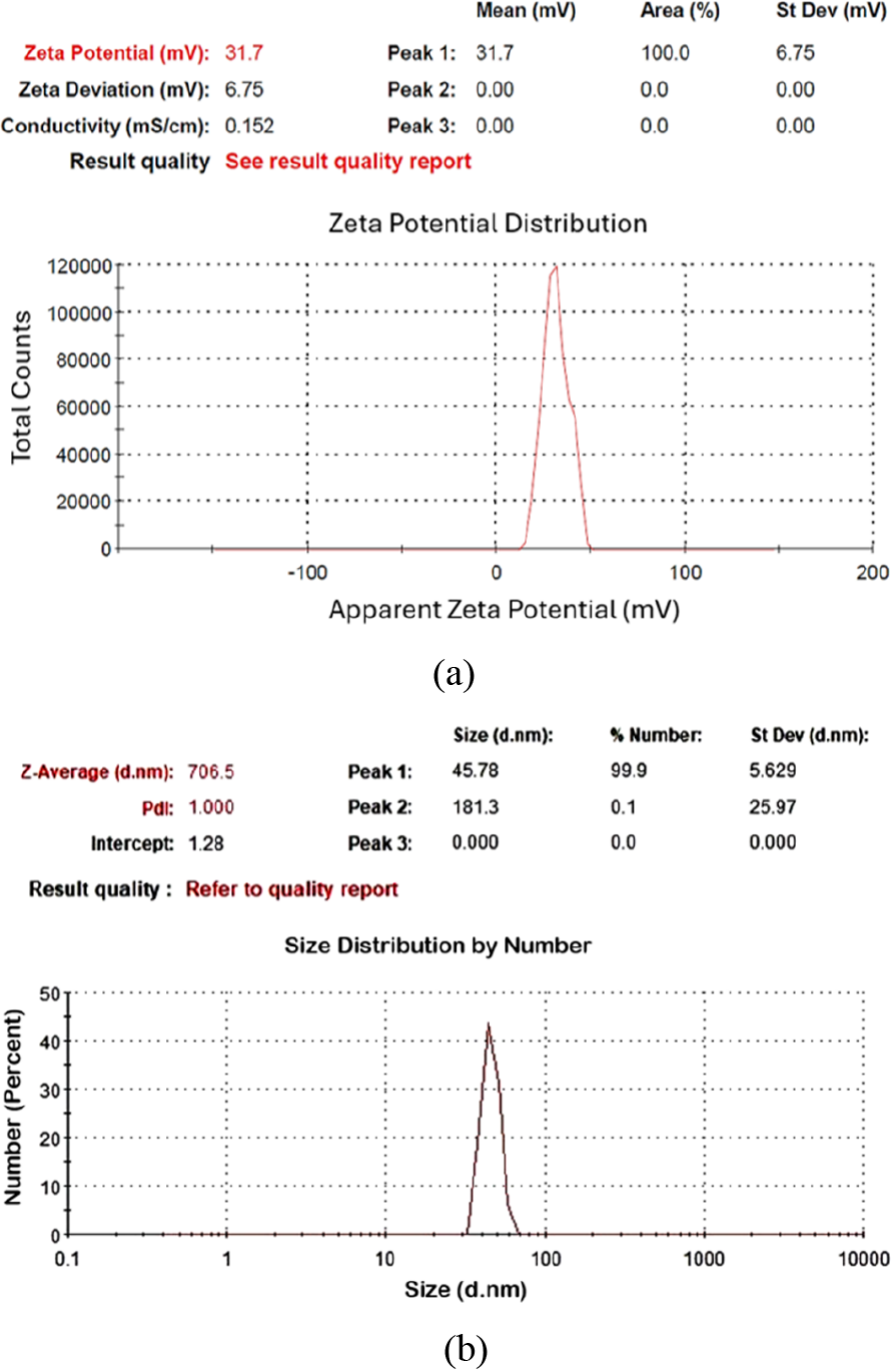
Analysis results of C-90–2m-6: (a) zeta potential, (b) particle size–number distribution.

**Table 3:** Characteristics of Colloids Prepared with Different Pulse Cycle Times with IP6

		particle size–number distribution		
colloid name	**V**_**ζ**_ (mV)	>100 nm (%)	particle size (d nm)	absorbance
C-10–2m-6	33.6	99.4	45.51	2.742
C-20–2m-6	14.8	99.9	59.09	2.567
C-30–2m-6	22.0	99.7	66.61	2.258
C-40–2m-6	27.3	99.8	59.58	2.685
C-50–2m-6	28.9	99.9	50.03	2.567
C-60–2m-6	27.8	97.3	44.06	2.329
C-70–2m-6	35.2	99.9	49.51	2.589
C-80–2m-6	26.0	99.9	110.1	2.43
C-90–2m-6	31.7	99.9	45.78	2.41
C-100–2m-6	26.5	100	38.04	2.31

### 3.3 Composition Analysis of Magnesium Hydroxide Nanocolloids

XRD, TEM, and EDS were used to analyze the crystalline structure, particle shape, particle size, lattice line width, and composition of C-70–2m-6. Figure 12 presents the XRD pattern of C-70–2m-6, which shows distinct diffraction peaks at 2θ = 18.38°, 37.83°, 50.93°, and 58.78°. These peaks are characteristic of crystalline magnesium hydroxide, confirming that the prepared colloid consists of Mg­(OH)_2_ nanoparticles.^8,37^

**Figure 12:**
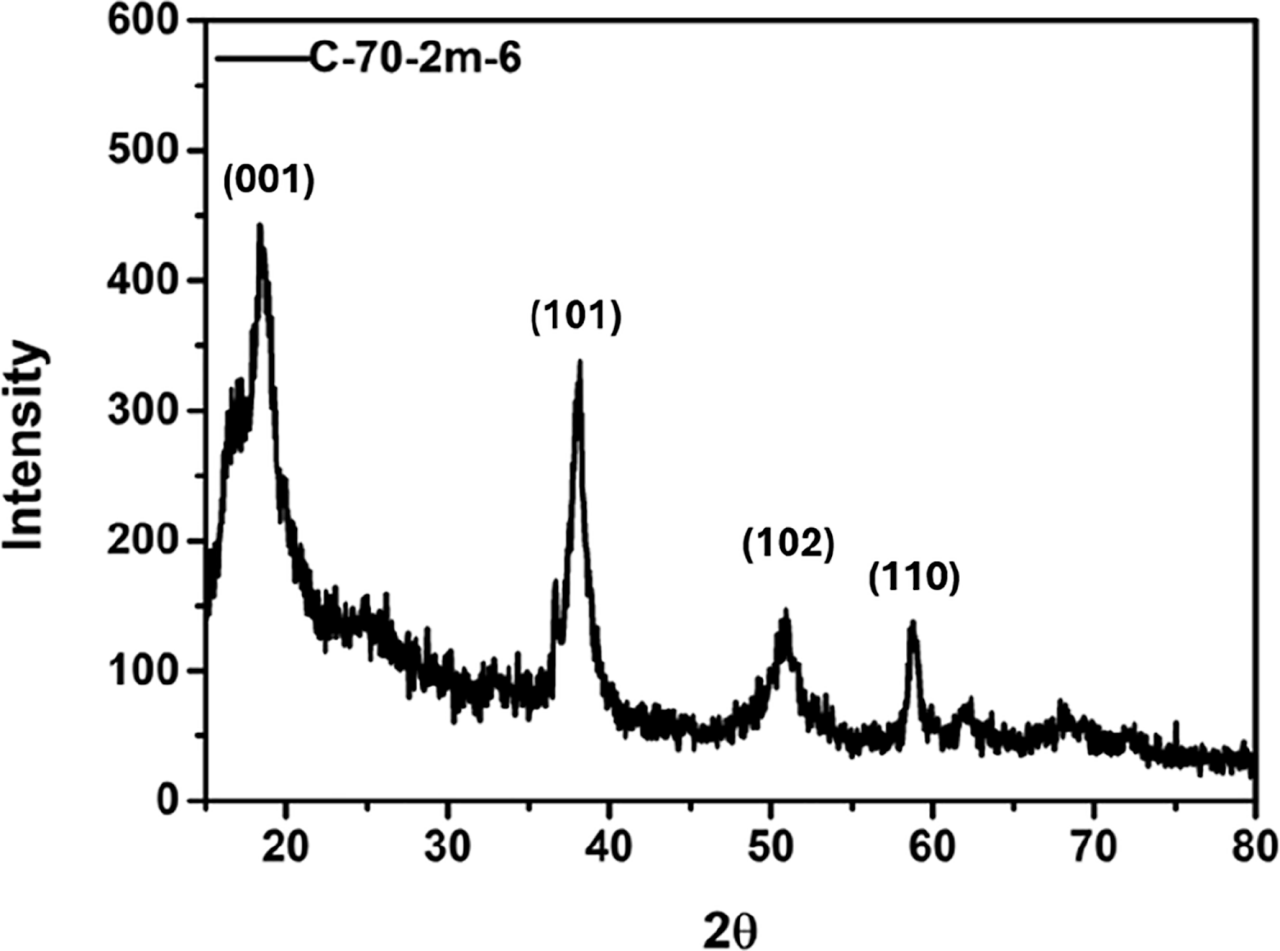
XRD diffractogram of C-70–2m-6.

Figure 13 shows different TEM images of C-70–2m-6. Figure 13a is a 60,000× magnified image at position A, demonstrating a large number of nanoparticles in the colloid. Figure 13b is a 100,000× magnified view of the region marked in Figure 13a, demonstrating nearly spherical nanoparticles. Figure 13c is a 300,000× magnified view of the region marked in Figure 13b, demonstrating nanoparticles with a diameter of approximately 87.6 nm, although no distinct lattice lines are visible. Figure 13d is an 800,000× magnified TEM image at position B, demonstrating nanoparticles with a diameter of approximately 36.921 nm, with alternating black and white lines representing lattice lines on particle surface. Figure 13e presents a Digital Micrograph image of the lattice fringes in Figure 13d. The total width of all 10 lattice lines was measured to be 2.37 nm, resulting in an average lattice width of 0.237 nm. This lattice spacing can be indexed to the (101) plane of crystalline Mg­(OH)_2_, which is consistent with the XRD diffraction peak observed at approximately 2θ≈38° in Figure 12.^8^ Figure 14 depicts the EDS spectrum of C-70–2m-6. The results indicate that the prepared colloid contains only magnesium and oxygen, confirming that it is a magnesium hydroxide nanocolloid.

**Figure 13:**
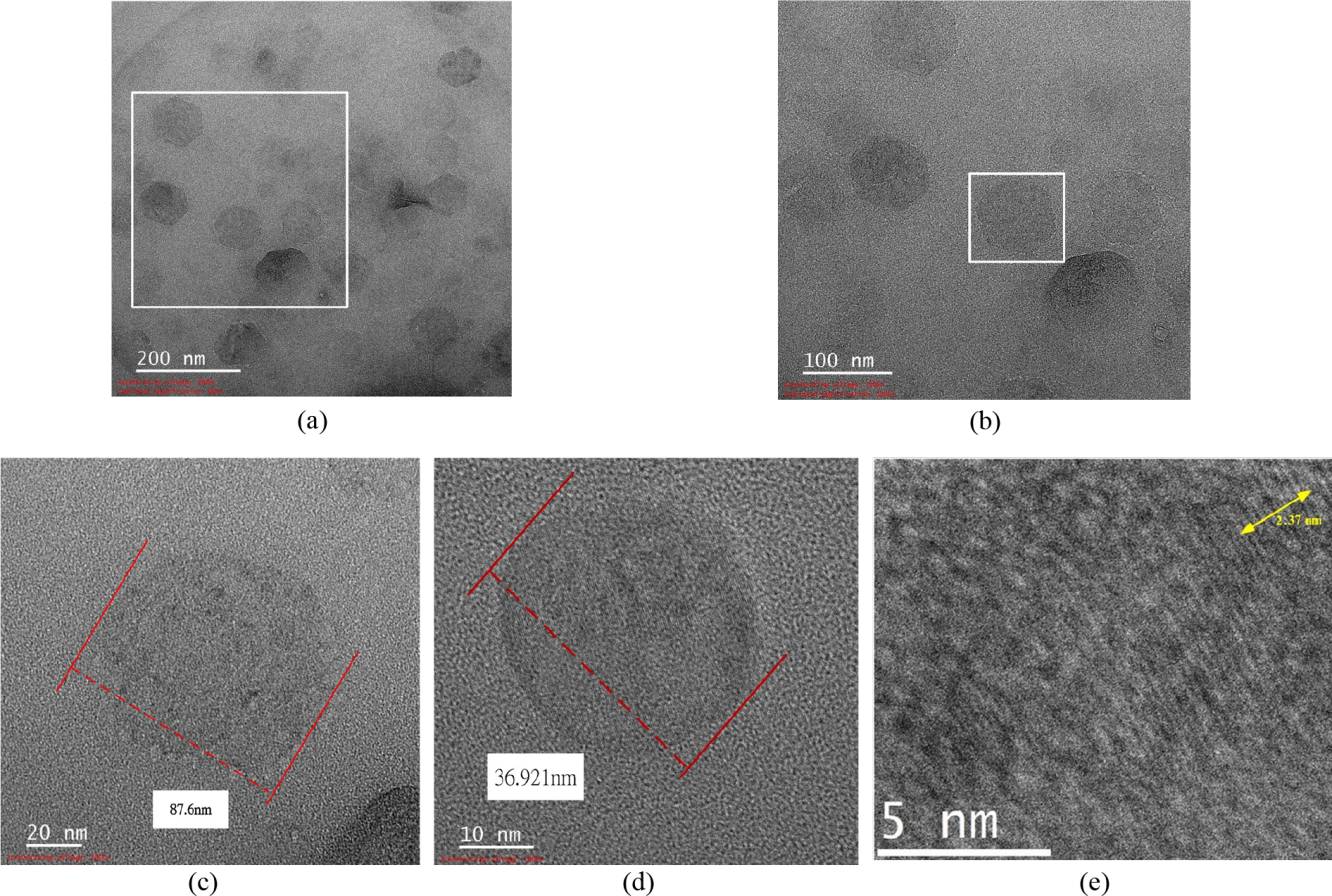
TEM images of C-70–2m-6: (a) position A magnified at 60,000×, (b) position A magnified at 100,000×, (c) position A magnified at 300,000×, (d) position B magnified at 800,000×, and (e) position B magnified at 1,600,000×.

**Figure 14:**
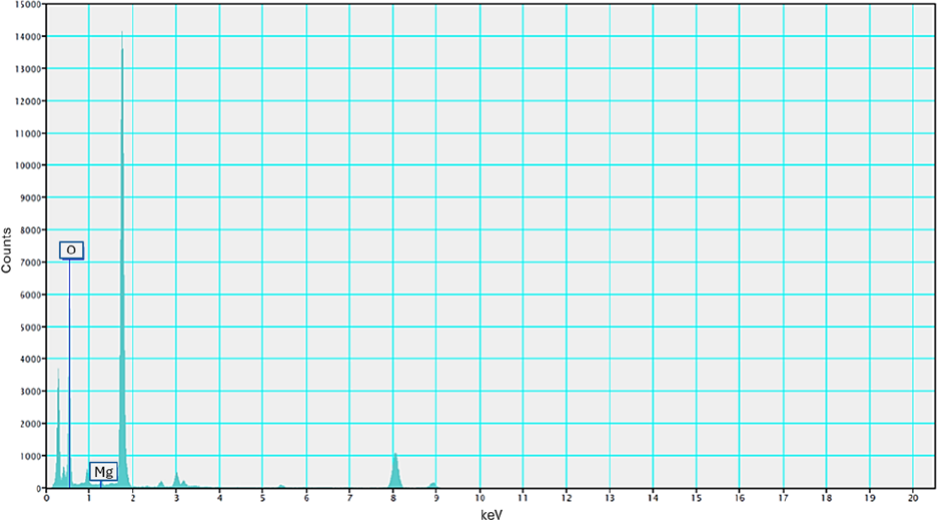
EDS spectra of magnesium hydroxide nanocolloids with a *T*_on_–*T*_off_ value of 70–70 μs.

The average crystallite size of the prepared Mg­(OH)_2_ nanocolloid was estimated using the Scherrer equation based on the major XRD diffraction peaks, as summarized in Table 4, where 2θ denotes the Bragg diffraction angle, fwhm represents the full width at half-maximum of the diffraction peak, and the crystallite size corresponds to the coherent diffraction domain size calculated from these parameters. The calculated crystallite sizes are on the nanometer scale, with an average value of approximately 8 nm.

**Table 4:** Crystallite Size Estimation of Mg­(OH)_2_ Using the Scherrer Equation

peak index	2θ (°)	fwhm (°2θ)	crystallite size (nm)
(001)	18.47	1.47	5.5
(101)	37.99	1.12	7.5
(102)	50.81	2.09	4.2
(110)	58.78	0.63	14.6
**average**			**≈ 8.0**

This crystallite size is smaller than the particle sizes observed by TEM and DLS, which is expected, since a single nanoparticle may consist of multiple nanocrystalline domains. Therefore, the XRD, TEM, and DLS results provide complementary information and consistently confirm the nanoscale characteristics of the prepared Mg­(OH)_2_ colloids.

### 3.4 Discharge Efficiency of Magnesium Hydroxide Nanocolloids

To determine the efficiency of discharge during colloid synthesis, an oscilloscope (Tektronix TDS 2024B) was used to measure the voltage and current in the IEG during the preparation process of magnesium hydroxide nanocolloids. Figure 15 depicts the values of voltage and current over five cycles in the IEG during the preparation of C-70–2m-6, with each cycle performed at a *T*_on_–*T*_off_ setting of 70–70 μs. Of these five cycles, T1, T3, and T4 exhibited successful discharge, with a voltage of 15 V and a current of 25 A during spark discharge, resulting in power consumption of 375 W. The duration of *T*_on_ was calculated as the sum of spark discharge time and *t*_D_. Because these cycles had a *t*_D_ value of almost zero, the duration of *T*_on_ was considered the actual spark discharge time. Among the three cycles with successful discharge, T4 had the longest *t*_D_, during which the voltage was approximately 130 V. After *t*_D_, the DF in the IEG was broken down, leading to a low-resistance state, which in turn caused a rapid increase in IEG current. By contrast, T2 and T5 exhibited unsuccessful discharge, with a voltage of 130 V in the IEG during *T*_on_ and a current of 0 A, indicating that the IEG remained in an open-circuit state during these cycles.

**Figure 15:**
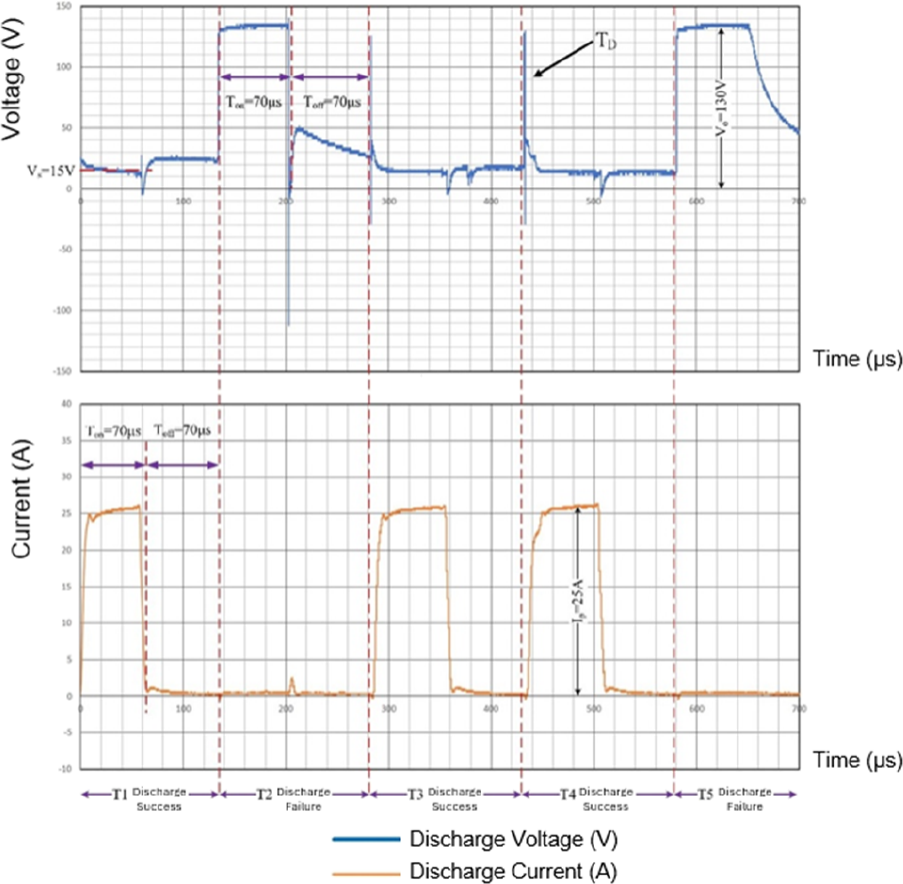
Voltage and current during the ESDM-based preparation of C-70–2m-6.

Discharge success rate (DSR) was defined as the ratio between the cumulative number of successful discharge cycles and the total number of cycles allowed for discharge. This rate was calculated using eq 2, where *N*_T–D_ is the cumulative number of successful discharge cycles and *N*_T–total_ is the total number of cycles allowed for discharge. Eqs 3 and 4 were used to calculate the total accumulated spark discharge time (*t*_total_) and total consumed energy (*W*_total_), respectively, where *D* is the duty cycle ratio of the pulse power supply during colloid preparation, *t*_prep_ is the colloid preparation time, and *P*_dis_ is the power when spark discharge is successful:

$$\mathrm{DSR}=\frac{N_{T-D}}{N_{T-\mathrm{total}}}$$ 2

$$t_{\mathrm{total}}=D\times t_{\mathrm{prep}}\times \mathrm{DSR}$$ 3

$$W_{\mathrm{total}}=P_{\mathrm{dis}}\times t_{\mathrm{total}}$$ 4

As shown in Figure 15, when *N*_T–D_ was 3 and *N*_T–total_ was 5, a DSR of 60% was obtained. During the preparation of C-70–2m-6, *t*_proc_ was 120 s, *D* was 0.5, and *P*_dis_ was 375 W. In C-70–2m-6, at a DSR of 60%, *t*_total_ was 36 s and *W*_total_ was 13,500 J. When the DSR reached 100%, C-70–2m-6 was found to consume the maximum energy, with *t*_total_= 60 s and *W*_total_ = 22,500 J. These results confirmed that the energy consumed in the IEG during the preparation of C-70–2m-6 was below 22,500 J.

The unsuccessful discharge cycles (e.g., T2 and T5 in Figure 15) can be attributed to an incomplete dielectric breakdown within the interelectrode gap. Transient factors, such as the accumulation of microbubbles generated from hydrogen evolution, residual molten debris from previous discharges, or localized variations in dielectric strength, may temporarily increase the insulation level of the dielectric fluid. Under such conditions, the applied voltage is insufficient to initiate plasma channel formation, resulting in an open-circuit state during the pulse-on period.

### 3.5 Discussion

In this study, the ESDM was used to synthesize magnesium hydroxide nanocolloids with 7 IP and 10 pulse cycle time settings, with a fixed preparation time of 2 min. In both colloid preparation experiments, in which the *T*_on_–*T*_off_ and IP settings were varied, C-70–2m-6 was found to exhibit optimal colloidal properties, with an IP setting of IP6 and a *T*_on_–*T*_off_ setting of 70–70 μs. The discharge current significantly influenced nanoparticle formation. At lower discharge current settings (e.g., IP1), the discharge energy and localized temperature were insufficient to vaporize the magnesium electrode material effectively. Under such conditions, the material remained predominantly in a molten state rather than undergoing vaporization and rapid quenching, which resulted in electrode adhesion and ineffective nanoparticle formation. This observation indicates that a sufficiently high discharge current is required to promote material vaporization and subsequent nanoparticle nucleation during the ESDM process.

The absorbance of C-70–2m-6 was calculated as 2.589, with a *V*_ζ_ value of 35.2 mV. TEM analysis indicated that the particles were nearly spherical, with the smallest particle diameter being approximately 36.921 nm. EDS analysis confirmed that the colloid contained only magnesium and oxygen, and XRD analysis verified that its crystalline structure was consistent with magnesium hydroxide.

## 4 Conclusions

In this study, magnesium hydroxide nanocolloids were successfully synthesized using the ESDM under ambient temperature and pressure conditions. These results confirmed the capability of the ESDM to produce magnesium hydroxide nanocolloids with small particle sizes and excellent suspension stability. Compared with conventional chemical precipitation and hydrothermal methods, which often require surfactants, stabilizers, or pH control to maintain colloidal stability, the ESDM achieved a zeta potential of 35.2 mV without the use of any chemical additives. The particle sizes obtained in this study (minimum ≈36.9 nm by TEM and average crystallite size ≈8 nm by XRD) fall within the typical nanoscale range reported for chemically synthesized Mg­(OH)_2_. However, unlike multistep chemical routes, the ESDM enables one-step synthesis under ambient temperature and pressure conditions, reducing process complexity and potential secondary contamination. Because the preparation and collection processes of this colloid were entirely conducted in deionized water, no nanoparticle dispersion occurred in the processing environment. This feature distinguishes the ESDM from conventional chemical synthesis routes, which may involve chemical additives and may pose potential environmental or occupational exposure risks.

The following are the main contributions of this study:

ESDM was successfully used to synthesize magnesium hydroxide nanocolloids with a nanoscale particle size and excellent suspension stability. No nanoparticle dispersion was observed in the preparation environment, confirming that ESDM addresses the limitations of chemical methods in the preparation of magnesium hydroxide nanocolloids.
The colloid synthesized in this study contained only hydrogen, oxygen, and magnesium, confirming its safety for use in biomedical settings and in environmental pollution control, with no concerns regarding adverse effects caused by additional elements.
Compared with other synthesis methods, ESDM offers a simpler process, produces a more chemically pure colloid, and enables a faster fabrication process. These advantages indicate the competitiveness of ESDM in the production of magnesium hydroxide nanocolloids.
An oscilloscope was used to measure the energy consumption in the IEG during the ESDM-based preparation process. The results indicated that the energy consumption of the C-70–2m-6 colloidal sample was below 22,500 J. This result demonstrates that the efficient synthesis of stable Mg­(OH)_2_ nanocolloids can be achieved without excessive energy input.
